# Scrub Typhus: Historic Perspective and Current Status of the Worldwide Presence of *Orientia* Species

**DOI:** 10.3390/tropicalmed5020049

**Published:** 2020-04-01

**Authors:** Allen L. Richards, Ju Jiang

**Affiliations:** 1 Department of Preventive Medicine and Biostatistics, Uniformed Services University of the Health Sciences, Bethesda, MD 20814, USA; 2 Henry M. Jackson Foundation for the Advancement of Military Medicine, Bethesda, MD 20817, USA; jjiang@HJFresearch.org

**Keywords:** scrub typhus, *Orientia* species, Tsutsugamushi Triangle

## Abstract

Scrub typhus and its etiological agents, *Orientia* species, have been around for a very long time. Historical reference to the rickettsial disease scrub typhus was first described in China (313 AD) by Hong Ge in a clinical manual (Zhouhofang) and in Japan (1810 AD) when Hakuju Hashimoto described tsutsuga, a noxious harmful disease in the Niigata prefecture. Other clinicians and scientists in Indonesia, Philippines, Taiwan, Australia, Vietnam, Malaysia, and India reported on diseases most likely to have been scrub typhus in the early 1900s. All of these initial reports about scrub typhus were from an area later designated as the Tsutsugamushi Triangle—an area encompassing Pakistan to the northwest, Japan to the northeast and northern Australia to the south. It was not until the 21st century that endemic scrub typhus occurring outside of the Tsutsugamushi Triangle was considered acceptable. This report describes the early history of scrub typhus, its distribution in and outside the Tsutsugamushi Triangle, and current knowledge of the causative agents, *Orientia* species.

## 1. Early History of Scrub Typhus and the Etiologic Agents of the Tsutsugamushi Triangle

### 1.1. Scrub Typhus Disease Presentation and Diagnosis

Scrub typhus, a febrile disease with mild to life-threatening manifestations, is characterized by rapid onset of fever, headache, chills, arthralgias and myalgias and often the presentation of eschar prior to and a macularpapular rash following initiation of disease [1,2,3,4,5,6]. The illness lasts approximately 3 weeks and ends without sequalae. Rapid response (24–72 h) to antibiotic treatment with tetracyclines, chloramphenicol, and azithromycin is characteristic and diagnostic [2,3,5,6]. In fatal cases, the disease is characterized by multi-organ failure, with pathologic lesions in lungs, kidneys, liver, and brain [3,5].

The lack of scrub typhus-specific signs and symptoms makes the clinical diagnosis very difficult [2,3,5]. Moreover, laboratory diagnosis at the time of illness is also very difficult, as antibodies do not reach detectable levels for 5–10 days after disease presentation, and the level of orientiae in the blood stream demonstrable by molecular methods only reaches detectable levels sporadically during acute illness and is unapparent after initial treatment with appropriate antibiotic treatment [7]. The specimen of choice, biopsy of eschar and/or rash, is unfortunately rarely obtained, though the level of *Orientia* DNA is in abundance, unaffected by prior antibiotic treatment, and maintained in the lesion for the life of the lesion [7].

### 1.2. Early History of Scrub Typhus

#### China

Historically, scrub typhus has been around for a very long time. Human historical reference to scrub typhus (Table 1) was first described in China’s Zhouhofang, a clinical manual, in 313 AD [8]. Subsequently, in 610, Yuan-Fang Chao described the epidemiology, clinical course, and treatment of the disease in a poem, which is considered medically accurate [8] and Shi-Zhen Li, a well-known physician, described the characteristics of the disease in a book entitled, “Ben Cao, Gang Mu” in 1596 [8]. In 1908, Ashburn and Craig reported that in China, “shashitsu,” the name for scrub typhus, occurred in old Chinese writings of more than a thousand years (Table 2). The authors also indicated that the Chinese recognized the disease as a distinct illness, and it was attributed to the bite of a mite which occurred in summer in certain districts that had been flooded by spring rains [1]. The red lice (sna ra) or mites had been associated with illness characterized by fever and a pustule at the site of injury. Moreover, they recognized that three days after the bite, high fever developed, and a pustule appeared at the site of the injury [1]. Clearly, the Chinese were well aware of scrub typhus (shashitsu) and for an extensive period of time.

#### Japan

In Japan, in 1810, Hakuju Hashimoto described “tsutsuga”, a noxious harmful disease in the Niigata prefecture of the main island of Japan [9]. However, according to Tanaka, as described in Ashburn and Craig [1], the name tsutsugamushi (tsutsuga = disease/illness and mushi (bug/insect)) has been around since the earliest historical times in Japan. Though reports of tsutsugamushi disease, from the northwest coast of the main island, Nippon (the two prefectures, Akito and Echigo, later changed to three prefectures, Akito, Yamagata and Niigata), and research associated with it was published in Japanese medical and science journals, it was not until Theobald Palm in 1878 [10] and Bälz and Kawakami in 1879 [11] that tsutsugamushi disease from Japan was reported in European journals.

#### The Etiology of Scrub Typhus in Japan

Up until the 1920s, the etiology of tsutsugamushi and, therefore, scrub typhus was unknown. In addition, multiple diseases which were later believed to be synonymous with tsutsugamushi were known as Japanese river fever, flood fever, island fever, Kedani (mite) disease, akamushi disease, shimamushi disease yochubio, and shashitsu [1] (Table 2). During the 1920s, the laboratories of Hayashi, Nagayo, Ogata, and Kawamura were working to discover the causative agent of tsutsugamushi. At this time, it was postulated that spirits, noxious air, parasites, bacteria, and viruses were possible causes of tsutsugamushi. In 1920, Hayashi indicated that the causative agent was a protozoan and named the agent *Theileria tsutsugamushi* [12]. However, by 1924, Hayashi indicated that the agent was not a protozoan, but most likely a rickettsia [13], as described by Kawamura et al. [8]. Hayashi, however, did not give his new agent a binomial name [8]. In the meantime, Nagayo demonstrated the causative agent of scrub typhus could be maintained in human and dog macrophages and that the agent could be passed to and cause disease in monkeys by intradermal and intracutaneous inoculations [14], as described by Kawamura [8]. In 1929, Ogata and Unno used a rabbit intratesticular inoculation technique to obtain the tsutsugamushi agent from a human blood sample and passed it to other rabbits (testis) [15]. This was the first time that the scrub typhus agent was isolated from a human following inoculation of the patient’s blood into rabbit testis and, subsequently, transferring the agent from that rabbit into another rabbit, showing the ability of the causative agent to be isolated and transferred [8]. Unfortunately, Ogata and Unno did not provide a binomial name for the agent in their report. However, they did provide the agent and the methodology to both Nagayo and Kawamura laboratories [8]. Ogata, subsequently, developed a new rabbit model for tsutsugamushi. This entailed infecting the anterior chamber of rabbits’ eyes. This proved to be a far more sensitive method of infection and, subsequently, Nagayo used this tsutsugamushi model in his laboratory [8]. Moreover, Nagayo utilized the agent from Ogata and the inoculation of the anterior chamber of rabbit eyes to grow a large number of rickettsiae. These organisms could, subsequently, be maintained in rabbit Descemet’s membrane cell cultures. The results and the proposed name for the agent of tsutsugamushi, *Rickettsia orientalis,* was reported in 1930 in the Japanese Journal of Experimental Medicine [16], as described by Kawamura et al. [8]. Ogata and Kawamura, also utilizing Ogata’s agent, reported on the etiology of tsutsugamushi in the German journal Zentralblatt für Bakteriologie in the same issue in 1931, naming the agent as *Rickettsia tsutsugamushi* and *Rickettsia akamushi*, respectively [17,18], as described by Kawamura et al. [8] (Table 3). In 1932, Hayashi concluded that his agent was the same as Ogata’s *R. tsutsugamushi* and Nagayo’s *R. orientalis* [19]. Further, also in 1932, Ogata reported a new laboratory animal model for tsutsugamushi—the intraperitoneal (IP) inoculation of mice for the growth of *R. tsutsugamushi* [20], as described by Kawamura et al. [8]. This is a scrub typhus laboratory animal model that is still used today [21]. In the 6th edition of Bergey’s manual, the name of the agent for scrub typhus was reported as *O. tsutsugamushi*. Though much controversy was associated with this name [8], it was not completely resolved until 1995 when *R. tsutsugamushi* was moved out of the genus *Rickettsia* and into its own genus *Orientia*, with the new species name, *Orientia tsutsugamushi* [22] (Table 3).

#### Indonesia

During the early 1900s, other clinicians and scientists in Asia, Australia, and Islands of the Indian and Pacific Oceans reported on local diseases most likely to have been scrub typhus (Table 2). Dr. Schüffner of Deli, Sumatra, Indonesia, described a disease, pseudotyphoid, that resembled scrub typhus as early as 1902 [23]. Later, he indicated that this disease was similar to Kedani fever (later determined to be scrub typhus) in Japan [24]. Subsequently, scrub typhus was discovered to be endemic for many islands throughout the Indonesian archipelago [25,26,27,28,29,30].

#### Taiwan

In 1908, Japanese clinicians reported that eastern Taiwan had a febrile disease with a rash that was reported as a tsutsugamushi disease-like ailment and was confirmed in 1914 to be a tsutsugamushi disease but with a lower fatality rate (approximately 3%), which was significantly different to the high mortality rate (~20–40%) seen with tsutsugamushi in Japan at this time [31]. Scrub typhus continues to be associated with the main island of Taiwan [32,33] as well as the highly endemic Pescadores Islands of the South China Sea [34,35,36].

#### The Philippines

Further, also in 1908, two cases of tsutsugamushi disease were described for two US military personnel, stationed at Camp Connell, Samar, the Philippines, based upon clinical records [1]. Ashburn had just returned from Japan where he had seen many cases of tsutsugamushi that Japanese clinicians had shown him prior to reviewing the clinical records for these two cases and reporting about the cases and tsutsugamushi disease [1]. During the repatriation of the Philippines in WWII, 284 cases of scrub typhus occurred in the month of November, 1944 [37,38]. One of the isolates from a US soldier deployed to the Guinan region of Samar Island, Volner strain, was used to develop a lyophilized rat lung-spleen vaccine. This was the first US scrub typhus vaccine ever tested in a field trail, that was unfortunately unsuccessful [39]. Subsequently, studies showed evidence of scrub typhus throughout the Philippines [37,40].

#### Australia

In 1910, Mossman fever, later described as endemic glandular fever and, subsequently, determined to be scrub typhus, was reported in North Queensland, Australia [41,42,43,44]. The endemic region of scrub typhus in Australia now includes Queensland [45,46], the islands of the Torres Strait [46], the Northern Territory [47], and western Australia [48,49].

#### Vietnam

In 1915, a fever of unknown etiology among two individuals was reported in Saigon, Vietnam [50] to be a disease similar to that described in Deli, Sumatra (i.e., pseudotyphoid), that was later determined to be scrub typhus [24]. Subsequently, the presence of scrub typhus throughout Vietnam was confirmed [38,51,52,53,54,55,56,57].

#### Korea

In 1915, a “mild” rickettsial disease called paratyphus was reported among 15 patients (no deaths) in the spring of 1913–1914 from Jemulpo, Incheon, Korea by Weir, a medical missionary [58]. Because only mild disease presentations with no deaths were associated with paratyphus, it was thought not to be epidemic typhus, which was endemic for Korea at the time. Moreover, the disease only developed during March–June and, in retrospect, it was assumed not to be murine typhus (seen year round and most commonly in the fall) or tsutsugamushi disease (seen in summer and fall). Thus, Chung and Kang believe it could have been Brill–Zinsser disease and not scrub typhus [59].

During the period of 1910–1945, there was some evidence of endemic scrub typhus in Korea. A study of mites attached to wild rats collected in Suwon were similar to *Trombicula akamshi*. In addition, rickettsial diseases with mild presentations and low OX19 titers may not have been murine typhus but scrub typhus [59]. During Japanese occupation, the Japanese physicians considered tsutsugamushi disease a severe disease with a 15–60% mortality rate. Thus, they may have overlooked a milder form of scrub typhus in Korea. In addition, the Weil–Felix test with OXK was not often utilized [59]. Following 1945, evidence increased for the presence of scrub typhus in Korea [59] and consequently the endemicity of scrub typhus became obvious by detection of infected mites, rodents and humans [60,61,62,63]. In recent years, there has been an ever-increasing number of scrub typhus cases reported in South Korea (2637 cases in 2001 to 10,485 cases in 2013) [59,64].

#### Malaysia

In 1900, the Institute of Medical Research (IMR) in Kuala Lumpur, Malaysia, was established as the Pathological Institute with the aim to promote the health status of the local population. In 1924, the institute began research on “tropical typhus” in the Federated Malay States. In the annual IMR Bulletin in 1925, Fletcher and Lesslar described tropical typhus as containing two components—an urban or shop typhus and a rural or scrub typhus [65].

Due to the fortuitous change in the composition of the Weil–Felix test, “tropical typhus” could be divided into two unique diseases. The Weil–Felix test initially utilized the strain of *Bacillus proteus* X.19 (*Proteus vulgaris*) that was isolated from the urine of a patient with epidemic typhus [66]. The *P. vulgaris* agent was not the cause of the disease but was found to be agglutinated by antibodies developed during epidemic typhus. The cross-reactivity of the antibodies to the *P. vulgaris* antigens (OX19) has been successfully used since 1916 to serologically diagnose epidemic typhus and murine typhus. Subsequently, another strain of *P. vulgaris* (OX2) was identified that reacted with the sera of spotted fever patients [7]. These patients also reacted to the OX19 to varying degrees. A third agglutinin (OXK) was, subsequently, identified in 1926 [67]. It was the *Proteus mirabilis* Kingsbury strain which reacted with sera from scrub typhus patients but not with sera from typhus or spotted fever patients [68,69]. With that new development, Fletcher and Lessar concluded that the rural tropical typhus or scrub typhus was the same as or similar to tsutsugamushi and unique from urban or shop typhus and other rickettsial diseases [70,71]. Moreover, it was determined that the urban or shop typhus form of tropical typhus was clinically the same as Brill’s disease and had the same Weil–Felix results. This disease was later referred to as murine typhus and the causative agent identified as *Rickettsia typhi* [72]. Throughout the subsequent history of the IMR scrub typhus, research continued with major advances in *O. tsutsugamushi* isolations, diagnostics, treatment/prophylaxis, vaccine and immunology research, and vector and ecology research [38]. Scrub typhus research is not limited to the IMR as indicated by recent publications [73,74,75,76,77,78].

#### India, Burma, Ceylon, and the Maldives

In 1932, Christian reported OXK-positive typhus cases that he believed were due to tick bites [79]. Due to the serologies conducted, they were most likely the first cases of scrub typhus reported from India. Similarly, in 1934, scrub typhus (OXK+) was reported among personnel from Simla Hills, India [80]. An investigation by Mehta reported the presence of *Trombicula deliensis* on rodents and shrews in the Simla Hills, suggesting that similar to the reports from Malaya, that these mites may be the vectors of scrub typhus [81]. Boyd reported on the presence of typhus among 110 cases in 1935, utilizing clinical presentations and Weil–Felix OXK serologies [82]. Interestingly, none of the OXK-positive cases in India presented with eschars [83]. In 1944, a report of two outbreaks of scrub typhus, which occurred during the period of 1937–1938 (n = 11) and 1939–1942 (n = 30), that were confirmed by Weil–Felix serology also indicated no presence of eschars [84]. One case from Boyd’s 110 cases was an individual who was OXK+ from Burma [82]. Subsequently, a study by Maitra and Sen Gupta showed the presence of scrub typhus and murine typhus (OXK and OX19 positive, respectively) in Burma [85]. The famous prototype, *O. tsutsugamushi* Gilliam, was contracted by Dr. Gilliam in 1944 on the equally famous Stillwell Road, in Burma [67]. Nicholls reported OXK-positive cases of tsutsugamushi (rural typhus) in nearby Ceylon [86]. Similarly, in Maldives, outbreaks of scrub typhus among British troops struck during the period of 1941–1944 [87]. Scrub typhus outbreaks occurred again in the period of 2002–2003 among the inhabitants of the Maldives, indicating the endemic nature of this disease [88]. This is certainly the case for India, where numerous publications have shown the breadth of scrub typhus throughout the subcontinent [89,90,91,92,93].

### 1.3. The Tsutsugamushi Triangle

**Figure 1:**
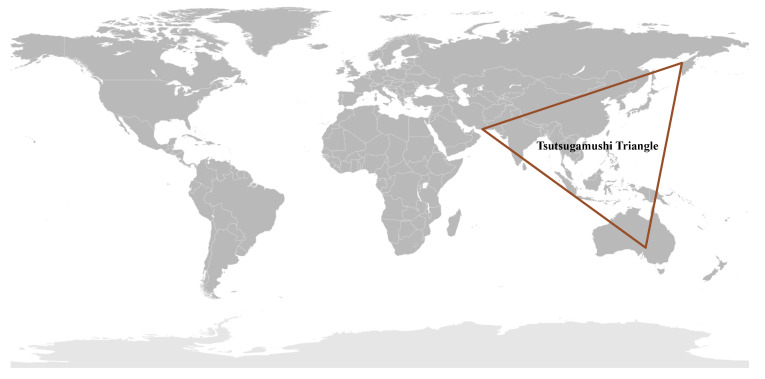
The Tsutsugamushi Triangle: Geographical Distribution of Scrub Typhus Caused by *Orientia tsutsugamushi.*

All of these reports of scrub typhus or diseases very similar to them throughout the Asia–Pacific region prior to WWII led to the assumption of a single rickettsial disease for a very large endemic region where many people were at risk of disease. Unfortunately, this assumption of a very large endemic area of scrub typhus was reinforced during WWII, where approximately 18,000 cases occurred among the allied forces and a similar number among the Japanese forces in the islands of Ceylon, Maldives, New Britain, Goodenough, and the Schouten Islands, and in the countries of China, Thailand, Japan, Australia, Lao, Cambodia, Vietnam, and Taiwan [25,37,38,83]. Contemporary reviews indicated the extent of scrub typhus distribution throughout the Tsutsugamushi Triangle [4,6,83,94,95,96,97,98], which included countries in the west (Pakistan, Afghanistan, Tajikistan, Nepal, India, Bangladesh, Sri Lanka, and Maldives), northeast (China, Russia, Republic of Korea, Japan, and Taiwan), south (Australia, Papua New Guinea, Indonesia, and the islands of the southwestern Pacific), and middle (Myanmar, Thailand, Laos, Cambodia, Malaysia, Vietnam, and Philippines) (Figure 1).

**Figure 2:**
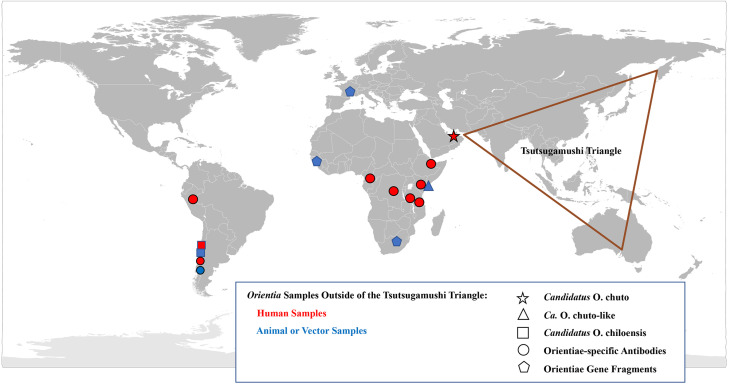
Geographical Distribution of *Orientia* spp. and the Scrub Typhus: A Worldwide Disease.

Consistent within the Tsutsugamushi Triangle has been the presence of a single species of *Orientia* which was identified within human cases, vector mites and mammalian hosts [8,83,94,98]. This species, *O. tsutsugamushi,* has a diversity of antigenic phenotypes and genetic genotypes found not only between countries but within countries [94,99,100]. However, as early as 1951, reports suggesting that scrub typhus occurred outside of the Tsutsugamushi Triangle began to emerge (Figure 2).

## 2. Scrub Typhus Outside the Tsutsugamushi Triangle

### 2.1. Case Investigations

#### Africa

In 1951, Giroud and Jadin published a report that indicated that scrub typhus occurred outside the Tsutsugamushi Triangle. This report described an outbreak of a febrile disease among native Africans from Ruanda-Urundi working on constructing a factory building in Musha Hill, Belgian Congo (now Rwanda and Burundi) [101]. To investigate the outbreak, the authors utilized tests/reagents available at the time to determine whether the illness was due to rickettsiae or coxiella infections. Due to the existence of *R. orientalis* (*O. tsutsugamushi*) antigens, the authors included scrub typhus in the panel of rickettsial diseases to assess. Among the ill Africans, several were positive for the various rickettsial antigens, including two individuals who reacted to the *R. orientalis* antigens. To confirm the skin tests, blood from the two *Orientia*-positive patients were tested for complement-fixing antibodies to *O. tsutsugamushi* and were found to be positive, with titers of 80 and 320. To assess the reactivity to the scrub typhus assays in other populations who lived closely with native Africans in Musha Hill, healthy individuals, including nine people born in Muscat, Oman, five born in Bombay, India, and two born in Africa, with parents who were born in Bombay, were tested using the same skin and blood tests for evidence of previous *O. tsutsugamushi* infection. The authors considered Muscat and Bombay as scrub typhus-endemic regions and thought that people from those areas may be antibody positive to *O. tsutsugamushi* and may, therefore, act as positive controls. Of the nine individuals, originally from Muscat, three had strong, weak, and negative skin reactivity and antibodies against *O. tsutsugamushi* were detected with titers of 1280 (four individuals) and 640 (three individuals) in eight. Of the five people born in Bombay, three displayed positive skin reactivity to *O. tsutsugamushi* antigens and, interestingly, the two people whose parents were from Bombay, but who were born in and never traveled outside of eastern Africa, were also positive. These results suggested the presence of scrub typhus in eastern Africa. The authors indicated that a similar study they conducted among natives in western Africa showed that they were negative for evidence of scrub typhus [101].

In the 1990s, three case reports insinuated, but could not confirm, the presence of scrub typhus in Africa. The first described an individual from Japan visiting the Republic of Congo, who presented with fever six days after his return from Africa [102]. The disease was identified as scrub typhus, though the possibility could not be ruled out that the patient had contracted scrub typhus in Japan, within the Tsutsugamushi Triangle, during the six days prior to disease presentation. The second case involved a US missionary who visited Cameroon [103]. Within two weeks of his visit, the missionary noted a lesion on his leg and he had a fever and a rash three days later. Two weeks later, the missionary returned to the US and was, subsequently, admitted to a hospital in which he was treated for a rickettsial disease. He recovered within 24 h with doxycycline treatment and he had a four-fold rise in titer from 256 to 1024 of antibodies against *O. tsutsugamushi*. Unfortunately, no molecular or culture evidence confirmed the case of scrub typhus. The third case was of an individual who had visited Tanzania [104]. She had noted a lesion on her right foot and had a three-day history of fever and headache after returning to the Netherlands. Her acute and convalescent sera showed a seroconversion against *O. tsutsugamushi* antigens from <16 to 1024 by an indirect immunofluorescence assay (IFA). Regrettably, none of these three cases had produced a culture of *Orientia* or had molecular evidence of the causative agents, thus, they were considered presumptive scrub typhus cases.

These results collectively implied that scrub typhus was endemic to Africa (Table 4) and the first study similarly implied the presence of scrub typhus in Arabia, both locations outside the Tsutsugamushi Triangle. Nevertheless, this notion of scrub typhus outside the Tsutsugamushi Triangle was not generally accepted and, therefore, scrub typhus was still considered an Asian-Australian-Pacific disease [94].

#### United Arab Emirates

**Figure 3:**
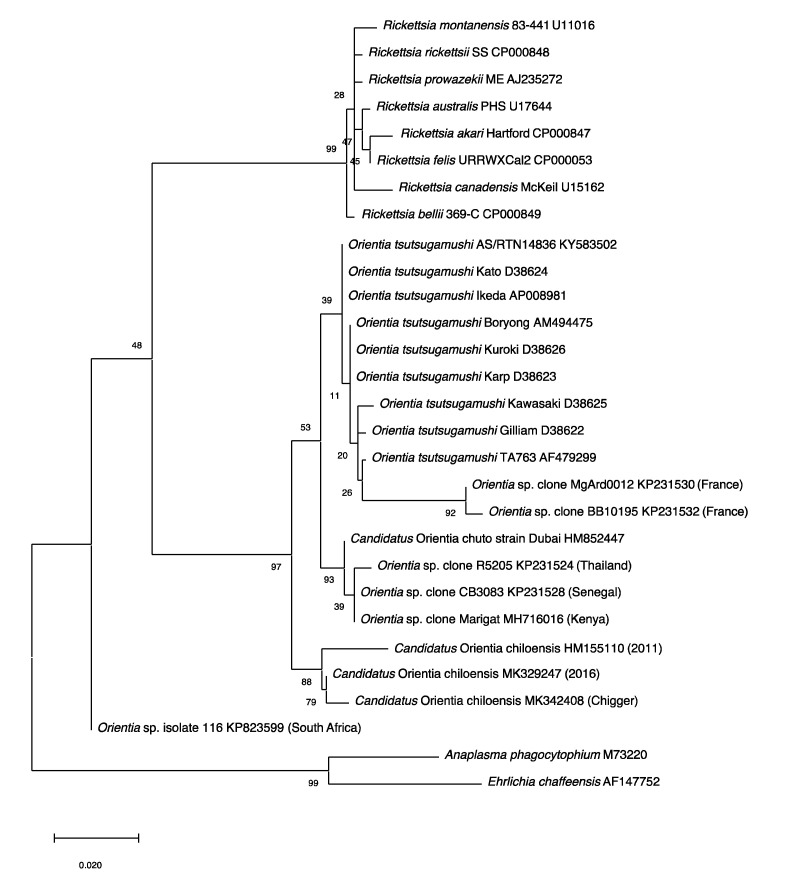
Phylogenetic Tree. The evolutionary relationships of *Orientia* species detected outside of the Tsutsugamushi Triangle were compared with *Orientia tsutsugamushi* strains, *Rickettsia* species and other bacteria. The tree was constructed with the 560 bp *rrs* gene fragments using the Maximum Likelihood method and the Tamura–Nei model in MEGAX. The values of the bootstrap test (1000 replicates) were shown next to the branches.

Conversely, the concept of scrub typhus as endemic outside of the Tsutsugamushi Triangle was firmly established with a 2010 report of a case contracted in Dubai, United Arab Emirates [105]. Not only was there serological evidence, but also molecular evidence and a culture of the infecting agent (Figure 3). The data substantiated the presence of scrub typhus on the Arabian Peninsula and the existence of a unique *Orientia* species other than *O. tsutsugamushi* (Table 5). The incompletely characterized agent has the proposed name of *Candidatus* Orientia chuto [105].

#### Chile

Subsequently, in 2011, a report of a scrub typhus case in Chile appeared that proved the existence of scrub typhus worldwide [106].

Unfortunately, an isolate was not recovered, though the limited molecular characterization of the *Orientia* DNA indicated, like the *Ca.* O. chuto, that this *Orientia* was not *O. tsutsugamushi* or for that matter *Ca.* O. chuto [106] (Figure 3).

### 2.2. Serological Evidence of Scrub Typhus Outside the Tsutsugamushi Triangle

Following these two astounding reports, investigators looked to confirm and expand upon these results, utilizing current serological and molecular assays for evidence of *Orientia* spp. infections outside of the Tsutsugamushi Triangle. Serological assays previously used for detecting rickettsial diseases outside of the Tsutsugamushi Triangle did not include those for scrub typhus group orientiae (STGO). Similarly, molecular assays for orientiae were not used to investigate the presence in mammalian hosts and/or arthropod vectors of orientiae outside of the Tsutsugamushi Triangle. This paradigm was changed with the two reports of scrub typhus in the United Arab Emirates and Chile. Thus, scientists included scrub typhus serological and molecular assays to conduct surveillance studies of rickettsial diseases in areas of Africa, South America, and Europe (Table 4). This resulted in the substantiation of scrub typhus outside of the Tsutsugamushi Triangle. In 2015, the first of these reports was of a seroprevalence study of fever patients from hospitals in Kenya. It was determined that 70 of 1401 (5%) patients had antibodies against *O. tsutsugamushi* that was confirmed by Western blot assays [107]. In an unrelated investigation conducted in western Kenya among sick children, paired acute and convalescent serum samples were tested, and it was determined that 15 of 281 patients (5.8%) had antibodies against *O. tsutsugamushi* ELISA antigens and 10 of these children seroconverted to *O. tsutsugamushi* antigens (3.6%). The seroreactivity was confirmed by Western blot analysis [108]. In Djibouti, a 20 week serosurvey of abattoir workers was conducted to determine their exposure to infectious disease agents. From multiple serum samples, it was ascertained that 3 of 49 workers had antibodies against *O. tsutsugamushi* ELISA antigens and one individual who reported a history of a febrile disease during the period of the study seroconverted to *O. tsutsugamushi* antigens by ELISA, IFA and Western blot tests [109].

### 2.3. Molecular Evidence of Scrub Typhus Outside the Tsutsugamushi Triangle

In addition to the serological data, molecular evidence for the presence of orientiae in Africa was conveyed in three separate reports (Figure 3). In 2015, Cosson et al. reported the presence of *Orientia* DNA among tissues of rodents from West Africa and Europe [110]. In South Africa, DNA preparation from the blood of a healthy dog from Mpumalanga Province had a 16S rRNA sequence that was 96.1% (247/257 bp) similar to that of *Orientia* spp. [111]. In East Africa, a rodent survey was conducted in a village where individuals who resided there had tested positive for antibodies against *O. tsutusgamushi*. Trombiculid mites were collected from the trapped rodents to assess them for molecular evidence of *Orientia*. DNA preparations provided evidence of *Orientia* species from sequences of gene fragments of the *rrs* and *htrA* that were most closely aligned to but not identical with *Ca.* O. chuto (Figure 3; Table 5) [112].

### 2.4. Endemic Scrub Typhus in South America

Two serological surveys were conducted in South America for rickettsial agents. The first, in Peru, assessed the role of rickettsial diseases in fever patients in the city of Iquitos on the Amazon river. It was determined that of 1124 individuals enrolled in the febrile surveillance study, 60 (5.3%) were seropositive against *O. tsutsugamushi* ELISA antigens and one person had a four-fold rise in titer, which suggested that he had scrub typhus. The ELISA results of this sample were confirmed by IFA [113]. The second survey involved a cross-sectional survey of dogs from Chiloé Island, the initial scrub typhus focus center in Chile [106,114]. It was revealed that of 202 dogs surveyed, 43 (21.3%) had immunoglobulin gamma (IgG) antibodies against *O. tsutsugamushi* antigens, with higher prevalence levels among dogs from rural areas and older dogs, and it was reported that dogs are a good sentinel animal for scrub typhus [115].

Since the initial scrub typhus case reported in 2011 [106], additional cases of scrub typhus (n > 40) [116] have been described from Chiloé Island [114,117] and from continental Chile [117,118]. The agents have been molecularly very much the same (Figure 3) from all cases from Chile [119], except for an imported case from the Republic of Korea, which was determined to be *O. tsutsugamushi* [120]. This is quite surprising when considering the extreme variation seen among the *O. tsutsugamushi* found throughout the Tsutsugamushi Triangle [94,99,100]. Similarly, characterization of orientiae from trombiculid mites of the genus *Herpetacarus* from Chiloé Island found the same orientiae as that associated with scrub typhus cases [121]. Thus, the molecular characterization of the agents both from human eschar/blood samples and trombiculid mites suggest a new scrub typhus agent, *Candidiatus* Orientia chiloensis (Figure 3; Table 5) [119,121].

The conservation of genetic variation in Chile orientiae may be related to the limitations placed on identifying cases and characterizing the agents—for the most part, utilizing clinical characteristics to identify cases (e.g., fever, headache, and eschar) and serological and molecular assays based upon *O. tsutsugamushi* antigens and sequences. Thus, as we identify more unique cases and develop better assays that are more sensitive and more generous in recognizing rare antibodies and sequences, with time, the antigenic and genetic variability of Chile orientiae may be discovered to be greater than current discoveries.

## Figures and Tables

**Table 1:** Early History of Scrub Typhus.

Year	Location	Book or Individual
313	China	Zhouhofang by Hong Ge
610	China	Yuan-Fang Chao
1596	China	Ben Cao Gang Mu by Shi-Zhen Li
1810	Japan	Hakuju Hashimoto
1902	Indonesia	Wilhelm Schüffner
1908	Philippines	P.M. Ashburn and C.F. Craig
1908	Taiwan	J. Hatori
1910	Australia	O. Smithson
1915	Vietnam	F. Noc and P. Gautron
1915	Malaysia	A. Kawamura Jr., H. Tanaka, A. Tamura
1932	India	C.R. Christian

**Table 2:** Synonyms for Scrub Typhus.

Synonyms	Country
Shashitsu	China
Tsutsugamushi	Japan
Kedani disease	Japan
Japanese river fever	Japan
Flood fever	Japan
Island fever	Japan
Akamushi disease	Japan
Shimamushi disease	Japan
Pseudotyphoid	Indonesia
Chigger-borne rickettsiosis	Ubiquitous
Mite-borne typhus	Ubiquitous
Mite fever	Ubiquitous
Rural or “K” form of tropical typhus	Malaysia
Fiévre exanthématique avec ulcére primaire	French Indo-China
Indian mite typhus	India
Mijtekoorts	Indonesia
Mossman fever	Australia
Sarina fever	Australia
Tropical typhus	Malaysia
Rural typhus	French Indo-China

**Table 3:** Previous and Current Names of the Scrub Typhus Agent from the Tsutsugamushi Triangle.

Previous Agent Names	Country	Reference
*Theileria tsutsugamushi*	Japan	[12]
*Rickettsia orientalis*	Japan	[16]
*Rickettsia tsutsugamushi*	Japan	[17]
*Rickettsia akamushi*	Japan	[18]
*Orientia tsutsugamushi*	Japan	[22]

**Table 4:** Evidence of Scrub Typhus Outside of the Tsutsugamushi Triangle.

Locations	Evidence
Belgian Congo, Africa	Outbreak investigation
Republic of Congo, Africa	Case report
Cameroon, Africa	Case report
Tanzania, Africa	Case report
Dubai, United Arab Emirates	Case report
Chiloé Island, Chile, South America	Chiloé cases; continental cases; dog serosurveillance; molecular characterization of cases; detection of agent in mites and rodent tissues
Kenya	Seroprevalence study; seroconversion cases; and molecular surveillance among mites and rodents
Europe and West Africa	Rodents tissue surveillance
Djibouti, Africa	Seroprevalence/conversion among abattoir workers
South Africa	Dog blood
Iquitos, Peru, South America	Seroprevalence/conversion among fever patients

**Table 5:** Current Known and Proposed Agents of Scrub Typhus.

Agent	Location
*Orientia tsutsugamushi*	Tsutsugamushi Triangle
*Candidatus* O. chuto	United Arab Emirates
*Ca.* O. chuto-like	Kenya
*Candidatus* O. chiloensis	Chile
